# Ascorbic acid can alleviate the degradation of levodopa and carbidopa induced by magnesium oxide

**DOI:** 10.1002/brb3.2672

**Published:** 2022-06-17

**Authors:** Noriyuki Miyaue, Madoka Kubo, Masahiro Nagai

**Affiliations:** ^1^ Department of Clinical Pharmacology and Therapeutics Ehime University Graduate School of Medicine Tohon Ehime Japan; ^2^ Department of Neurology Saiseikai Matsuyama Hospital Matsuyama Ehime Japan

**Keywords:** ascorbic acid, carbidopa, levodopa, magnesium oxide

## Abstract

**Introduction:**

Levodopa and carbidopa are reported to be degraded by magnesium oxide (MgO), which is often used as a laxative for patients with Parkinson's disease (PD). Ascorbic acid (AsA) can stabilize levodopa and carbidopa solutions; however, the effect of AsA on the degradation of levodopa and carbidopa induced by MgO has not been fully investigated.

**Methods:**

The effect of AsA was evaluated using in vitro examinations, compared with lemon juice, and by measuring the plasma concentration of levodopa in a patient with PD.

**Results:**

In vitro experiments showed that the relative concentrations of levodopa remained almost constant, and the relative concentrations of carbidopa decreased with time with addition of MgO. AsA mitigated this effect in a concentration‐dependent manner, whereas the addition of lemon juice caused little change, although the pH decreased to the same extent. The results of levodopa pharmacokinetics of the patient showed that the area under the plasma concentration‒time curve values from hour 0 to 8 were 53.00 μmol·h/L with regular administration and 67.27 μmol·h/L with co‐administration of AsA.

**Conclusions:**

AsA can mitigate the degradation of carbidopa induced by MgO and may contribute to improving the bioavailability of levodopa in patients with PD.

## Graphical abstract

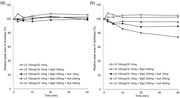

## 1 INTRODUCTION

Parkinson's disease (PD) is the second most common neurodegenerative disease. Levodopa is the most potent medication for the symptoms of PD and is typically co‐administered with peripheral l‐3,4‐dihydroxyphenylalanine (DOPA) decarboxylase inhibitors, such as carbidopa. Levodopa is absorbed mainly in the proximal small intestine, and its absorption is affected by various factors, including gastrointestinal motility, gastric acid secretion, and dietary protein content (Contin & Martinelli, 2010). Constipation is also considered as one of the factors and occurs in approximately two‐thirds of patients with PD (Pedrosa Carrasco et al., 2018). Although magnesium oxide (MgO) preparation is often used as a laxative for patients with PD, degradation of levodopa and carbidopa by MgO has been reported (Kashihara et al., 2019; Omotani et al., 2016). In patients with PD taking MgO for constipation, the absorption of levodopa and carbidopa may be reduced, resulting in lower plasma concentrations. As the disease progresses, the number of dopamine neurons in the substantia nigra pars compacta decreases, and presynaptic stores become depleted; consequently, the striatal dopamine concentration closely relates to the plasma concentration of levodopa (Wright & Waters, 2013). Therefore, reduced absorption of levodopa and carbidopa can lead to deteriorating clinical symptoms, especially in patients with advanced PD. In contrast, ascorbic acid (AsA) stabilizes levodopa and carbidopa solutions (Kurth et al., 1993; Pappert et al., 1996). However, the extent to which AsA can alleviate the degradation of levodopa and carbidopa induced by MgO has not been fully investigated.

In the present study, we investigated the effect of AsA on the degradation of levodopa and carbidopa using mixed suspensions with MgO in in vitro studies and by measuring the plasma concentration of levodopa in a patient with PD receiving levodopa preparations and MgO via a percutaneous endoscopic gastrostomy (PEG) tube.

## 2 MATERIALS AND METHODS

### 2.1 In vitro study

We evaluated the effect of AsA on the MgO‐induced degradation of levodopa and carbidopa in vitro. The following mixed suspensions were prepared with purified water (50 ml):

- 100 mg levodopa and 10 mg carbidopa.
- 100 mg levodopa, 10 mg carbidopa, and 250 mg MgO.
- 100 mg levodopa, 10 mg carbidopa, 250 mg MgO, and AsA (10, 200, or 500 mg).
- 100 mg levodopa, 10 mg carbidopa, 250 mg MgO, and 100% lemon juice (0.1 or 5 ml) including 2 mg AsA and 840 mg citric acid per 15 ml (POKKA SAPPORO Lemon 100^®^, POKKA SAPPORO Food & Beverage Ltd., Aichi, Japan).

After each solution was allowed to react at room temperature, time‐course changes in levodopa and carbidopa concentrations in the solutions were investigated (at 0, 2, 5, 10, 20, and 40 min). The collected sample (20 μl) at each point was diluted with purified water (200 μl) and filtered through a 0.45‐μm membrane filter. Aliquots (50 μl) were injected into the liquid chromatography‐mass spectrometry/mass spectrometry system to evaluate levodopa and carbidopa concentrations. Chromatographic separations were performed on an Agilent 1260 Infinity chromatographic system (Agilent Technologies, Santa Clara, CA, USA). The analytes were separated on a ZORBAX Eclipse Plus C18 (2.1 mm × 50 mm, 1.8 μm). The mobile phase consisted of 85% acetonitrile and 0.5% formic acid (95:5, v/v) at a flow rate of 0.2 ml/min. Mass spectrometric detection was performed using an amaZon speed ion trap mass spectrometer (Bruker Daltonics, Bremen, Germany) in the positive electrospray ionization mode. Quantitation was performed using multiple reaction monitoring transitions of m/z 197.9→181.0 for levodopa and m/z 226.8→181.0 for carbidopa.

Levodopa and carbidopa concentrations are expressed as a percentage of the starting concentration, which was 100. All experiments were performed in triplicates, except for the experiments with lemon juice, which were performed in duplicate. The data are presented as the mean.

### 2.2 Case study

The pharmacokinetics of levodopa was evaluated in an 86‐year‐old woman who was diagnosed with PD at the age of 70 years. She received a single levodopa/carbidopa tablet containing 100 mg of levodopa and 10 mg of carbidopa every 2 h and a single tablet containing 250 mg of MgO concomitantly every 4 h via a PEG tube as a suspension prepared in advance. Blood samples were collected every 30 min from the first levodopa administration on the day until 8 h later on 2 days: one in the condition with regular administration and the other with co‐administration of 200 mg of AsA. We measured plasma concentrations of levodopa by high‐performance liquid chromatography with electrochemical detection, as previously reported (Miyaue et al., 2021). Written informed consent was obtained from the patient.

## 3 RESULTS

**FIGURE 1:**
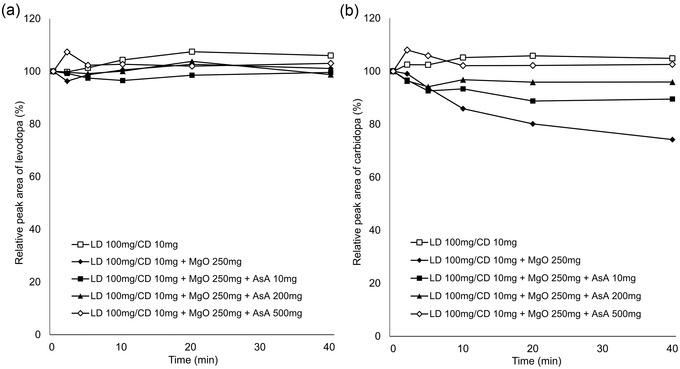
Time‐course change of levodopa (a) and carbidopa (b) concentrations in LD/CD suspension, LD/CD and MgO suspension, and LD/CD, MgO, and AsA (10, 200, 500 mg) suspension. LD, levodopa; CD, carbidopa; MgO, magnesium oxide; AsA, ascorbic acid

**FIGURE 2:**
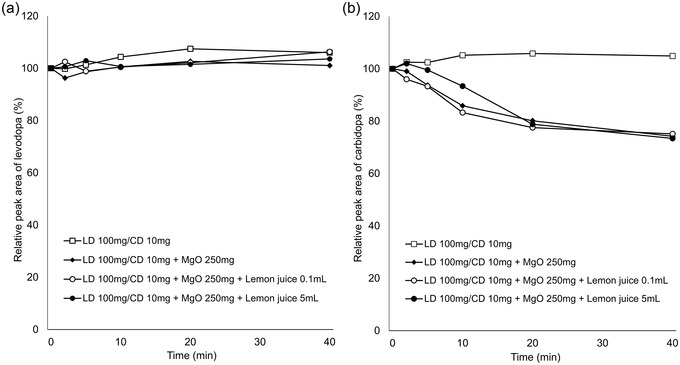
Time‐course change of levodopa (a) and carbidopa (b) concentrations in LD/CD suspension, LD/CD and MgO suspension, and LD/CD, MgO, and lemon juice (0.1, 5 ml) suspension. LD, levodopa; CD, carbidopa; MgO, magnesium oxide

In vitro experiments showed that the relative concentrations of levodopa remained virtually constant over the experimental period under all conditions (Figures 1a and 2a). In contrast, the relative concentrations of carbidopa decreased over time with addition of MgO. AsA mitigated this effect in a concentration‐dependent manner (Figure 1b), whereas there was almost no change with the addition of lemon juice (Figure 2b). The pH values of each solution at the final sampling point are listed in Table 1.

**TABLE 1:** The pH values at the final sampling point

		pH
LD 100 mg/CD 10 mg	(*n* = 3)	6.90 (0.21)
LD 100 mg/CD 10 mg + MgO 100 mg	(*n* = 3)	9.85 (0.28)
LD 100 mg/CD 10 mg + MgO 100 mg + AsA 10 mg	(*n* = 3)	9.59 (0.15)
LD 100 mg/CD 10 mg + MgO 100 mg + AsA 200 mg	(*n* = 3)	9.00 (0.22)
LD 100 mg/CD 10 mg + MgO 100 mg + AsA 500 mg	(*n* = 3)	8.46 (0.25)
LD 100 mg/CD 10 mg + MgO 100 mg + lemon juice 0.1 mL	(*n* = 2)	9.63 (0.17)
LD 100 mg/CD 10 mg + MgO 100 mg + lemon juice 5 mL	(*n* = 2)	8.81 (0.09)

*Note*: Values are shown as mean (standard deviation).

Abbreviations: LD, levodopa; CD, carbidopa; MgO, magnesium oxide; AsA, ascorbic acid.

**FIGURE 3:**
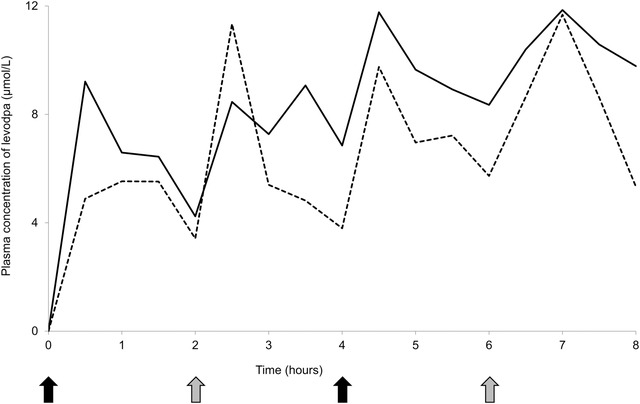
Plasma concentrations of levodopa following the administration of suspensions, including 100 mg of levodopa, 10 mg of carbidopa, and 250 mg of magnesium oxide (*black arrows*) and 100 mg of levodopa and 10 mg of carbidopa (*gray arrows*) via a gastrostomy tube with (*solid line*) or without co‐administration of 200 mg of ascorbic acid (*dotted line*)

The result of measuring plasma levodopa concentration of the patient is shown in Figure 3. The area under the plasma concentration‒time curve values from hour 0 to 8, calculated according to the linear trapezoidal rule, were 53.00 μmol·h/L with regular administration and 67.27 μmol·h/L with co‐administration of 200 mg of AsA. There was no evident difference in clinical symptoms between these conditions.

## 4 DISCUSSION

This study investigated the effect of AsA from in vitro examinations and levodopa pharmacokinetics in a patient with PD. This is the first study to show that AsA can alleviate the degradation of levodopa and carbidopa induced by MgO.

Gastric pH affects the dissolution of orally administered levodopa in the stomach because levodopa dissolves readily under acidic conditions and is easily oxidized in alkaline pH (Siddhuraju & Becker, 2001). In addition, levodopa produces dopamine through decarboxylation, whereas through oxidation, it produces melanin as the end product (Zhou et al., 2012). Our in vitro study demonstrated that co‐suspension with MgO led to decreased concentrations of carbidopa but stable concentrations of levodopa, which is consistent with the result of a previous study (Kashihara et al., 2019). In the previous study, the stability of levodopa and carbidopa was maintained in the pH‐adjusted MgO suspension, suggesting that the alkaline pH condition resulted in the degradation of carbidopa. Our data showed that MgO suspension reduced the concentrations of carbidopa by 26% at 40 min, but the addition of 10 mg of AsA mitigated the reduction to 11%, 200 mg to 5%, and 500 mg to the same level as the MgO‐free suspension (Figure 1). In contrast, lemon juice containing AsA had little effect on the degradation of carbidopa induced by MgO (Figure 2). The pH value of the solution containing 200 mg of AsA (9.00) was higher than that of the solution with 5 ml lemon juice (8.81), containing 0.67 mg of AsA, at the final sampling point (at 40 min). Therefore, we suggest that the differences in action on carbidopa were attributable to the difference in the content of AsA, not to the alkaline pH condition.

Levodopa pharmacokinetics in the patient demonstrated mild improvement with co‐administration of AsA (Figure 3). Although simultaneous plasma concentrations of carbidopa were not measured in the patients, based on the knowledge that DOPA decarboxylation is the major pathway of metabolizing levodopa and the results of in vitro experiments, this may be caused by the reduced carbidopa concentration. In our data, the effect of 250 mg of MgO on 10 mg of carbidopa was considerably mitigated by 200 mg of AsA, suggesting that the combination of AsA stabilized carbidopa in the patient. To date, only one study has investigated the effect of AsA on levodopa pharmacokinetics in patients with PD (Nagayama et al., 2004). The study showed that AsA can improve the absorption of levodopa in elderly patients with PD, showing poor levodopa bioavailability. In addition, a tablet containing 10 mg of carbidopa for every 100 mg of levodopa is used for the treatment of PD in Japan, whereas a levodopa preparation containing 25 mg of carbidopa is available outside Japan. Because a higher proportion of carbidopa was reported to increase the bioavailability of levodopa and improve the therapeutic effect (Kaakkola et al., 1985; Tourtellotte et al., 1980), the effect of MgO on carbidopa can be even greater for a levodopa preparation containing 25 mg of carbidopa. Furthermore, many patients with PD develop dysphagia during the disease course (Kalf et al., 2012), and some require PEG at an advanced stage. In patients receiving levodopa preparations suspended orally or via a PEG tube, the suspension of MgO together may reduce the amount of levodopa and carbidopa absorbed, resulting in lower plasma concentrations. AsA prevents levodopa from breaking down in the periphery and increases its bioavailability and absorption in the brain. Although excessive AsA intake may cause side effects, such as gastrointestinal upset and nausea, the maximum tolerated dose of AsA is 2000 mg/day (Hathcock et al., 2005), and the dose used in the present study (250 mg of AsA per 100 mg of levodopa) is considered safe.

AsA has important functions in the central nervous system, including antioxidant protection, peptide amidation, myelin formation, synaptic potentiation, and protection against glutamate toxicity (May, 2012). Previous studies have shown that plasma AsA levels in patients with PD were lower than those in healthy controls (Sudha et al., 2003) and that patients with severe PD had lower lymphocyte AsA levels (Ide et al., 2015). AsA may be effective in patients with PD for purposes other than the stabilization of levodopa and carbidopa.

This study had some limitations. First, we evaluated the effect of AsA on levodopa pharmacokinetics in only one patient with PD receiving levodopa preparations and MgO through a PEG tube and plasma concentrations of carbidopa were not measured. Further large‐scale studies, including measurement of carbidopa concentrations, are required to validate the effect of AsA on levodopa and carbidopa suspended with MgO in patients with PD. Second, this study was conducted with the carbidopa formulation, but not with benserazide, another DOPA decarboxylase inhibitor. Although both formulations contained the same amount of levodopa (100 mg), the benserazide formulation contained 25 mg of benserazide, whereas the carbidopa formulation contained 10 mg of carbidopa. The benserazide formulation was reported to have a higher maximum plasma concentration and area under the plasma concentration time curve than the carbidopa formulation (Iwaki et al., 2015), which may be more susceptible to MgO. Finally, the precise mechanism by which the addition of AsA ameliorates the MgO‐induced decrease in carbidopa concentration remains unclear in the present study. AsA has been identified to act as a catechol‐O‐methyltransferase (Bonifacio et al., 2007), and it has been suggested that carbidopa is metabolized by this enzyme (Vickers et al., 1975). Further studies, including analysis of metabolites, are required to clarify the effects of AsA on carbidopa.

In conclusion, we investigated the effect of AsA on the degradation of levodopa and carbidopa induced by MgO. Although MgO causes degradation of carbidopa, AsA can mitigate this effect and may contribute to improving the bioavailability of levodopa. Further studies are required to confirm the efficacy of AsA on levodopa pharmacokinetics in patients with PD using MgO preparations.

### PEER REVIEW

The peer review history for this article is available at https://publons.com/publon/10.1002/brb3.2672

## Data Availability

The data that support the findings of this study are available from the corresponding author upon reasonable request.
